# Comparative analysis of the transduction efficiency of five adeno associated virus serotypes and VSV-G pseudotype lentiviral vector in lung cancer cells

**DOI:** 10.1186/1743-422X-10-86

**Published:** 2013-03-14

**Authors:** Chiachen Chen, Victoria Akerstrom, James Baus, Michael S Lan, Mary B Breslin

**Affiliations:** 1 Research Institute For Children, Children’s Hospital New Orleans, New Orleans, LA, 70118, USA; 2 Diana Helis Henry Medical Research Foundation, New Orleans, LA, 70119, USA; 3 Department of Pediatrics and Biochemistry and Molecular Biology, LSUHSC, New Orleans, LA, 70112, USA

**Keywords:** Adeno associated virus, VSV-G pseudotyped lentivirus, Lung cancer, SCLC, NSCLC

## Abstract

**Background:**

Lung cancer is the leading cause of cancer-related deaths in the US. Recombinant vectors based on adeno-associated virus (AAV) and lentivirus are promising delivery tools for gene therapy due to low toxicity and long term expression. The efficiency of the gene delivery system is one of the most important factors directly related to the success of gene therapy.

**Methods:**

We infected SCLC cell lines, SHP-77, DMS 53, NCI-H82, NCI-H69, NCI-H727, NCI-H1155, and NSCLC cell lines, NCI-H23, NCI-H661, and NCI-H460 with VSV-G pseudo-typed lentivirus or 5 AAV serotypes, AAV2/1, AAV2/2, AAV2/4, AAV2/5, and AAV2/8 expressing the CMV promoter mCherry or green fluorescent protein transgene (EGFP). The transduction efficiency was analyzed by fluorescent microscopy and flow cytometry.

**Results:**

Of all the serotypes of AAV examined, AAV2/1 was the optimal serotype in most of the lung cancer cell lines except for NCI-H69 and NCI-H82. The highest transduction rate achieved with AAV2/1 was between 30–50% at MOI 100. Compared to all AAV serotypes, lentivirus had the highest transduction efficiency of over 50% at MOI 1. Even in NCI-H69 cells resistant to all AAV serotypes, lentivirus had a 10-40% transduction rate. To date, AAV2 is the most widely-used serotype to deliver a transgene. Our results showed the transduction efficiency of AAVs tested was AAV2/1 > AA2/5 = AAV2/2> > AAV2/4 and AAV2/8.

**Conclusions:**

This study demonstrated that VSV-G pseudotyped lentivirus and AAV2/1 can mediate expression of a transgene for lung cancer gene therapy. Overall, our results showed that lentivirus is the best candidate to deliver a transgene into lung cancer cells for treatment.

## Introduction

Lung cancer remains the most common cause of cancer-related death in USA and worldwide. In the United States, the number of lung cancer deaths exceeds the rates from breast, prostate, and colon cancers combined. There are two main types of lung cancer, non-small cell lung cancer (NSCLC) and small cell lung cancer (SCLC). NSCLC accounts for 80% of lung cancers and has a propensity to metastasize and to be resistant to chemotherapy and radiation therapy [1,2]. Gene therapy provides a new and promising treatment for those patients who have both chemo- and radiotherapy resistant relapse. One of the most important factors directly related to the success of gene therapy is the efficiency of the gene delivery system [3,4].

Adeno-associated virus, AAV, is emerging as one of the most popular gene delivery systems because of a lack of pathogenicity in humans, and its long term and efficient transgene expression in various cells. At least 11 serotypes of AAVs have been isolated from animal tissues and the cell tropism is different among the serotypes [5-8]. Although AAV2 is the most widely used serotype, in cells that lack the necessary cellular receptor components for infection, the transduction efficiency is limited. High titers of virus are required to achieve an effective level of transgene expression. Many studies suggest that replacement of the AAV2 capsid with the capsid from other AAV serotypes results in increased cell tropism [9]. The transduction efficiency of AAVs in lung cancer cells has not been extensively studied. One report showed that SCLC was completely refractory to AAV2 transduction due to a complete lack of the cell receptor on the cell surface [10]. Several studies indicated AAV1 and AAV5 were the optimal serotypes for infection of lung epithelial cells [11-13]. Analysis of nine serotyopes of AAV in mice showed that AAV4 resulted in the greatest number of genome copies in lung [7]. In this study, we used the recombinant AAV2 (rAAV) backbone and replaced the capsid gene with the AAV1, AAV4, AAV5, and AAV8 serotypes.

Lentiviral vectors have been proposed as an efficient gene delivery system for gene therapy. The vectors can achieve stable long term expression of the transgene and high transduction efficiency in various cells and tissue [14]. To increase the transduction efficiency and the cellular tropism of lentivirus, the lentiviral envelope is substituted by the vesticular stomatitis virus glycoprotein (VSV-G) because its phospholipid receptor is widely expressed in a variety of cells [15,16].

In this study, we compare the transduction efficiency of 5 serotypes of rAAVs with a VSV-G pseudotyped lentiviral vector expressing the fluorescent genes, mCherry or EGFP in 11 different lung cancer cell lines (both NSCLC and SCLC). Our results provide valuable information for choosing an efficient delivery system for lung cancer gene therapy.

## Results

### Transduction efficiency of 5 serotypes rAAV vectors in lung cancer cell lines

**Figure 1:**
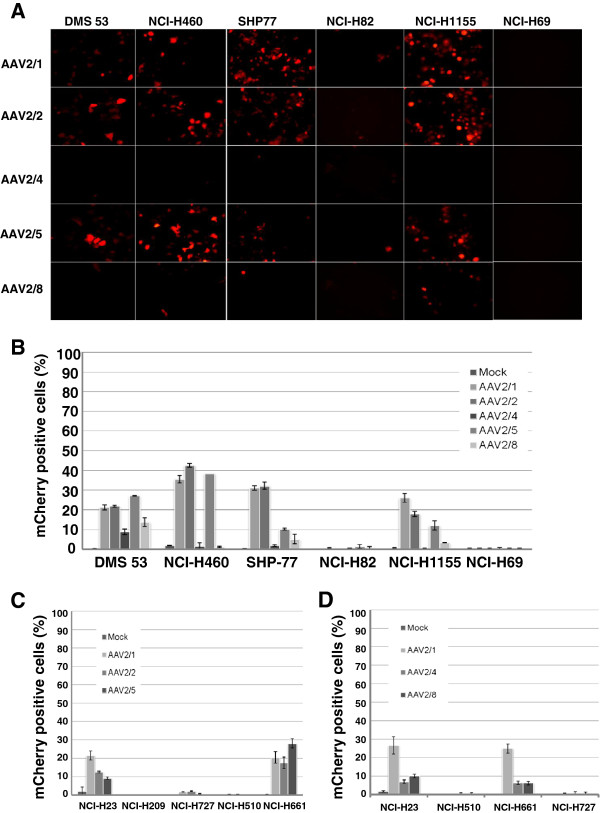
**Transduction efficiency of different serotypes of recombinant AAV virus in various human lung cancer cells lines.** Cells were infected with AAV2/1, AAV2/2, AAV2/4, AAV2/5 and AAV2/8 virus that carried the CMV-promoter driven mCherry transgene at a MOI 100. (**A**) mCherry expression was detected by fluorescence microscopy and transduction efficiency was measured by FACS analysis (**B,C,D**) on day 6 post-infection. Original magnification: 100X. The infection was done in triplicate on two separate occasions. Data are presented as the mean of positive cells ± SEM.

To compare the transduction efficiency of rAAV serotypes in lung cancer cells, six lung cancer cell lines, DMS 53, NCI-H460, SHP-77, NCI-H82, NCI-H1155 and NCI-H69 were infected with MOI 100 AAV2/1, AAV2/2, AAV2/4, AAV2/5 and AAV2/8 that carried the CMV promoter–driven mCherry transgene. On day 6 post-infection, mCherry expression was detected by fluorescent microscopy and FACS analysis. Seventy-two hours post- infection, a weak fluorescent signal was observed in the tranduced cells, SHP-77, NCI-H1155, NCI-H460 and DMS 53. The percentage of mCherry positive cells increased in a time-dependent manner (data not show). The results indicate that the lung cancer cells tested were highly resistant to AAV2/4 and AAV2/8 infection. Only DMS 53 cells, NCI-H23 and NCI-H661 were infected by the AAV2/4 and the AAV2/8 virus however the transduction rate was low (13%–6%) (Figure 1B, 1D, Table 1). Only a few SHP-77 and NCI-H1155 cells were infected by the AAV2/8 virus but the proportion was very low, only 5.1% and 3.3%. The NCI-H82 and NCI-H69 cell lines were completely refractory to all AAV serotypes tested (Figure 1B). The highest transduction efficiency achieved was with the NCI-H460 cells with AAV2/1, AAV2/2 and AAV2/5 and reached around 40% (Figure 1B, Table 1). Given the observation that AAV2/1, AAV2/2 and AAV2/5 gave the most promising transduction rates in NCI-H460, DMS 53, SHP-77 and NCI-H1155, we tested the transduction efficiency of AAV2/1, AAV2/2 and AAV2/5 (MOI of 100) in an additional 5 lung cancer cell lines, NCI-H23, NCI-H209, NCI-H727, NCI-H510 and NCI-H661 (Figure 1C). Due to the tropism variance that is seen between the different lung cancer cell lines, we also test the transduction efficiency using AAV2/4 and AAV2/8 in the additional cell lines, NCI-H23, NCI-H727, NCI-H510 and NCI-H661. The results indicated that those lung cancer cells were also resistant to AAV2/4 and AAV2/8 infection and the transduction rate was lower than 10% (Figure 1D). NCI-H209, NCI-H727, and NCI-H510 behaved similar to the NCI-H69 and NCI-H82 and were completely resistant to AAV infection (Figure 1C, 1D). The highest infection rate seen was with AAV2/5 in NCI-H661 at 28.7%. In the NCI-H23 NSCLC cells, the highest infection rate was with AAV2/1 at 21.5%. The expression of the transgene, mCherry or EGFP could be first detected at 72-hours post-infection with fluorescent microscopy and the signal persisted for 8 days. In all 11 lung cancer cell lines we tested, the comparison of the transduction efficiency of the 5 serotypes of AAV was AAV2/1 > AAV2/5 = AAV2/2> > AAV8 > AAV4.

**Table 1:** Infection rates of 5 serotypes of AAV in 11 lung cancer cell lines

**Cells**	**NCI-H23**	**DMS53**	**NCI-H69**	**SHP77**	**NCI-H82**	**NCI-H209**	**NCI-H460**	**NCI-H510**	**NCI-H661**	**NCI-H727**	**NCI-H1155**
**Serotype**											
**AAV2/1**	24.04 ± 3.54	21.25 ± 0.12	0.27 ± 0.04	31.08 ± 0.06	0.7 ± 0.06	0.03 ± 0.001	35.6 ± 1.83	0.08 ± 0.05	24.88 ± 2.46	1.63 ± 0.15	26.05 ± 2.2
**AAV2/2**	12.50 ± 2.42	21.9 ± 1.44	0.3 ± 0.047	32.18 ± 1.88	0.4 ± 0.06	0.033 ± 0.001	42.6 ± 1.80	0.26 ± 0.06	17.5 ± 2.5	2.1 ± 0.2	17.93 ± 1.33
**AAV2/4**	6.80 ± 2.04	8.8 ± 0.42	0.4 ± 0.08	1.9 ± 0.53	0.5 ± 0.06	ND	1.5 ± 1.02	0.33 ± 0.02	6.33 ± 0.94	0.49 ± 0.07	0.5 ± 0.06
**AAV2/5**	9.13 ± 0.51	27.13 ± 1.34	0.3 ± 0.041	10.25 ± 1.06	1.3 ± 0.1	0.03 ± 0.001	38.5 ± 1.03	0.06 ± 0.05	28.07 ± 3.2	0.67 ± 0.15	12.13 ± 2.29
**AAV2/8**	10.05 ± 1.8	13.8 ± 0.15	0.4 ± 0.02	5.1 ± 0.51	0.5 ± 0.07	ND	1.2 ± 0.15	0.013 ± 0.01	6.2 ± 1.2	0.37 ± 0.13	3.3 ± 0.06

Data are presented as the mean of positive cells ± SEM.

### Comparative analysis of transduction efficiency of rAAV and VSV-G pseudotyped lentiviral vector in NCI-H69 and NCI-H1155 cells

**Figure 2:**
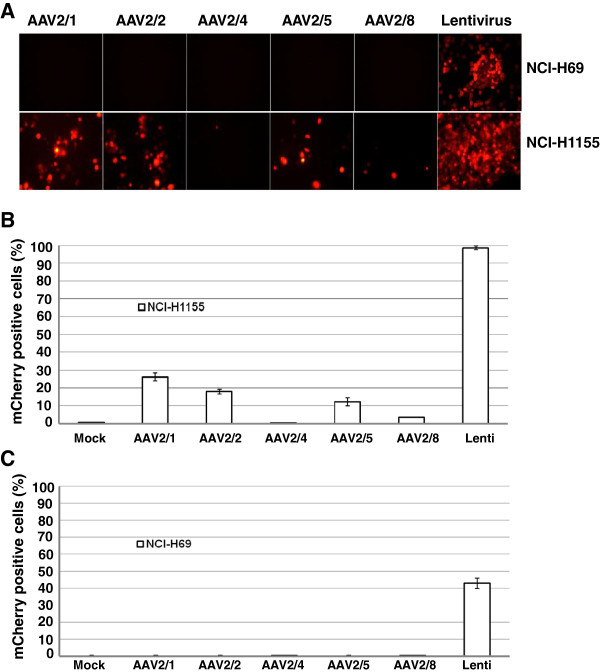
**Comparative analysis of transduction efficiency of rAAV and lentivirus in NCI-H69 and NCI-H1155.** Cells were infected with AAV2/1, AAV2/2, AAV2/4, AAV2/5, AAV2/8 (MOI 100) and lentivirus that harbor the CMV promoter driven mCherry fluorescent protein. At 6 days post-infection, the fluorescent signal was detected by fluorescent microscopy (**A**) and FACS analysis (**B,C**). Original magnification: 100X. (**B**) The proportion of mCherry positive cells in NCI-H1155 was markedly increased for the lentivirus in comparison to all of the serotypes of rAAVs utilized. (**C**) In NCI-H69, the strongest signal was observed with the lentivirus and all the rAAV virus were weaker. The infection was done in triplicate. Data are presented as the mean of positive cells ± SEM.

When infected with 5 different serotypes of AAV, NCI-H69 and H82 were completely refractory to infection. Less than 30% of NCI-H1155 cells were infected by rAAV1 and only 10–15% were infected with rAAV2/2 and rAAV2/5. To seek a more efficient viral delivery system, NCI-H69 and NCI-H1155 were infected with lentivirus at MOI of 100. The transduction efficiency was measured at 6 days post-infection by fluorescent microscopy and FACS analysis (Figure 2). As previously noted, NCI-H69 is highly resistant to infection by all the AAV serotypes tested (Figure 1, Table 1), while the transduction with the VSV-G lentivirus at the same MOI reached 42% (Figure 2A and 2B). For NCI-H1155 cells, AAV2/1–mediated transgene expression was 26%, highest among all the serotypes of AAV used (Figure 1B). NCI-H1155 cells were readily susceptible to lentivirus infection and the infection rate reached nearly 100% (Figure 2C).

### FACS analysis of eleven lung cancer cell lines cells with lentivirus at increasing MOIs

**Figure 3:**
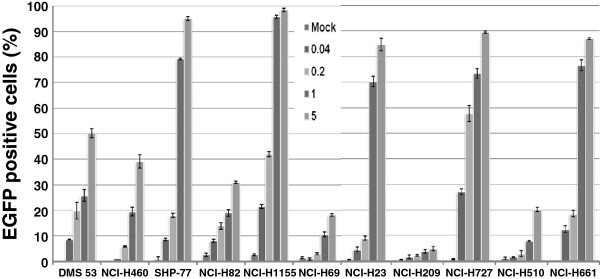
**The transduction efficiency of lentivirus in eleven human lung cancer lines at increasing MOIs.** A lentivirus containing the CMV promoter driven EGFP protein was infected into the human lung cancer cells at MOI 0.04-5. FACS analysis was performed on day 6. The infection was done in triplicate on two separate occasions. Data are presented as the mean of positive cells ± SEM.

Given the encouraging results seen with the NCI-H69 and NCI-H1155 cells, the panel of lung cancer cells were tested with lentivirus. The lung cancer cells were infected with a serial dilution, MOI 0.04-5 of lentivirus. Most cell lines were susceptible to lentivirus infection. At low MOI, MOI = 5, the transduction efficiency of the lentivirus in 7 out of 11 cell lines is around 40% or higher (Figure 3). In some of cell lines such as SHP-77, NCI-H1155, NCI-H23, NCI-H727, and NCI-H661 even at MOI 1, the infection rate is over 50% (Figure 3) compared to the highest transduction efficiency of rAAV (MOI 100) that reached a maximum of 40%. Even the most refractory cell lines NCI-H69 and NCI-H82 reached between 20–30% (Figure 1B and Figure 3). At MOI of 5, the transduction efficiency of the lentivirus in NCI-H510 and NCI-H727 was 19.8% and 89.9% respectively (Figure 3). The transduction efficiency of the lentivirus in the eleven lung cancer cell lines we tested was much better than any serotype of AAV. The transduction rate of the lentivirus reached or exceeded the AAV infection rates but at a much lower MOI.

### High transduction efficiency of lentivirus in the AAV-resistant human lung cancer cell lines

**Figure 4:**
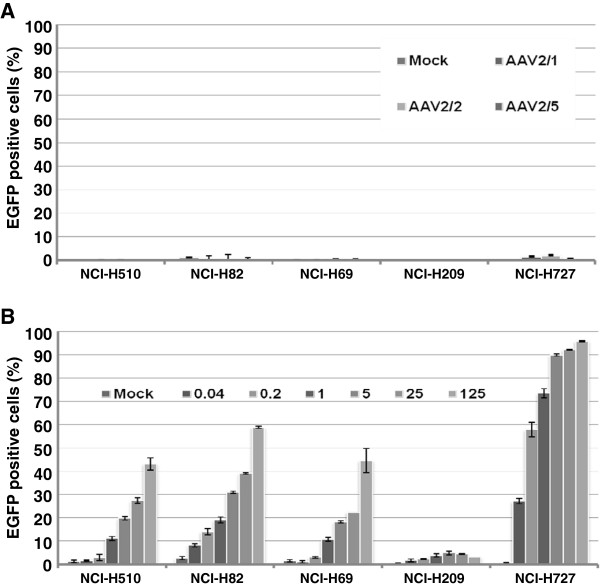
**Comparison of lentivirus transduction efficiency with AAVs in the AAV-resistant human lung cancer cell lines.** NCI-H510, NCI-H82, NCI-H69, NCI-H209 and NCI-H727 cells were infected with **A**) MOI 100 AAV2/1, AAV2/2 and AAV2/5 and compared to with **B**) lentivirus at increasing MOI 0.04-125. Except for NCI-H209 cells, the other lung cancer cell lines that were refractory to rAAVs viral transduction were susceptible to the lentivirus infection. The infection was done in triplicate on two separate occasions. Data are presented as the mean of positive cells ± SEM.

At low MOI, lentivirus could reach 85–100% in the NCI-H1155, SHP-77, NCI-H727, NCI-H661 and NCI-H23 cells. Despite this encouraging result, the H209 only reached 5%, H510 and H69 20% and H82 30% infection rates. Therefore, a comparison between MOI 100 of AAV2/1, AAV2/2, and AAV2/5 (Figure 4A) was done with a broader range of lentivirus between MOI 0.04-125 (Figure 4B). The transduction efficiency was detected using FACS analysis on day 6 post-infection. The results demonstrated that the transduction efficiency of the lentivirus was superior to all tested AAV serotypes. In contrast to AAV, the infection rate was more than 40% in most of the AAV-resistant cell lines except for NCI-H209 which was resistant to all the virus types tested (Figure 4). In NCI-H727, cells the infection rate of lentivirus reached nearly 100% compared to less than 5% with AAV virus (Figure 4). Overall, our results indicated that lentivirus is superior to rAAV as a gene delivery system for lung cancer gene therapy even at a low MOI.

## Discussion

Gene therapy is a modern and alternative treatment for classes of cancers that cannot be readily removed by surgery and are resistant to chemo- and radiotherapies. Adeno-associated virus and lentivirus have been widely studied and used for gene therapy. A high transduction efficiency of the chosen viral delivery vector plays an important role in the ultimate success rate of the gene therapy [3]. AAV is rapidly becoming one of the most popular gene delivery systems because of its long term expression and the large number of serotypes with the ability to transduce a diverse array of cell types. Numerous serotypes of AAV have been identified and each serotype has its own unique, specific cell tropism based on their different capsid proteins. There are at least 10 serotypes cloned and used for packaging the AAV2 ITR cassette to establish a recombinant AAV with the AAV2 genome and another serotype’s capsid protein thus expanding the cell and tissue tropism of the AAV2 virus [6,17,18]. In the present study, we compare the transduction efficiency of five different serotypes of rAAV, AAV2/1, AAV2/2, AAV2/4, AAV2/5 and AAV2/8 expressing the fluorescent gene mCherry or EGFP in a variety of lung cancer cell lines. In this study, the transduction efficiency of the AAV2/1, AAV2/5 and AAV2/2 virus is superior to other tested serotypes. The transduction efficiency in normal human airway epithelia was rAAV2/1> > rAAAV2/2 = rAAV2/5 and was different in mouse airway, rAAV2/5 > rAAV2/1> > rAAV2/2 [19]. Several studies indicated that AAV1 and AAV5 were more efficient at transducing murine airway, human airway epithelia and A549, a human adenocarcinoma lung cancer cell line [12,19,20]. A comprehensive study of AAV1-9 serotypes in isolated mouse tissues following systemic injection showed that AAV4 injection was localized to the thoracic cavity [7]. Luciferase activity assayed from the isolated mouse tissues showed AAV4 was highest in heart followed by lung. Although AAV4 showed luciferase activity in lung it was ten times lower than the level in the heart. AAV4 has the highest cellular tropism for muscle cells. The high AAV4 signal detected in the chest area was most likely due to the muscle and bone surrounding the lung [7]. Our results indicated that most lung cancer cell lines are highly resistant to AAV2/4 and AAV2/8. Though the transduction profiles of rAAV serotypes are different among the lung cancer cell lines, our result indicates that the transduction efficiency in lung cancer is rAAV2/1 > rAAV2/5 = rAAV2/2> > rAAV2/8 > rAAV2/4.

Five of the eleven lung cancer cell lines tested in this study were resistant to all five serotypes of AAV. The difference in lung cancer susceptibility to rAAV is most likely due to the expression of the viral receptor on the cell surface. The first step in AAV infection into the target cells is binding to the cell surface receptor. Five of the lung cancer cell lines, H69, H82, H209, H510, and H727 were completely refractory to all serotypes of AAV infection and likely lack the cellular receptor on the cell surface for virus binding. Unfortunately, these five lines do not all belong to the same lung cancer subtype. NCI-H69, H510, and H209 are classical SCLC cells, H82 are SCLC variant, and H727 are carcinoid. Therefore, the feasibility of using a single AAV serotype to treat even the same class of lung cancer is not likely. The knowledge about the cellular receptors for the different serotypes of AAV is not clear. Rohr *et al*. found that SCLC cells were resistant to AAV2 infection because they lack expression of the essential coreceptor FGFR-1 for viral internalization, αV-intergrin, and heparan sulfate proteoglycans [8,10,21]. AAV4 and AAV5 require different sialic acid-containing glycoproteins for binding to target cells. The α2,3 and α2,6 sialic acid on N-linked glycoproteins are required for AAV5 and AAV1 binding and transduction. The α 2,3 O-linked sialic acid glycoprotein is required for AAV4 binding to cells [21,22]. Some reports indicate that a 37/67-kDa laminin receptor is the cellular receptor for AAV8 virus [23]. The different gene sequences and the structure of the viral capsid protein determine how it interacts with the different cellular receptors and determine the different cell tropism for the AAV serotypes. Future research is needed to define the receptor of the different AAV serotypes to identify the one most suitable for use in SCLC cells.

For the success of gene therapy for human lung cancers, our investigation indicates that the VSV-G pseudotyped lentivirus is superior to the AAV serotypes investigated as a delivery system. With MOI 5, the transduction efficiency of the VSV-G pseudotyped lentivirus reached 50% in most of lung cancer cells as compared to the highest infection rate achieved with AAV which peaked around 30–40% at MOI 100. One report measured the transduction rate of AAV in the lung adenocarcinoma cell line, A549 which reached near 70%. However, the amount of AAV particles required to achieve this rate was MOI of 10^4^ to 5×10^4^. In a clinical setting, such a high concentration of virus is not feasible. Even at high MOI (MOI of 10^4^), the infection rate with AAV2/5 is less than 20% in A549 cells [20,24,25]. In contrast, in the cells refractory to AAV infection, the transduction rate of lentivirus at MOI 125 reached to more than 40%. In NCI-H727 cells, the transduction efficiency reached almost 100%. The result indicates that the tropism of lentivirus for lung cancer cells is broad. The VSV-G-pseudotyped lentivirus has been shown to not only stabilize the viral particles allowing concentration to high titers by ultracentrifugation, but also broaden the tissue and cell tropism. The phospholipid receptor for the VSV-G protein is expressed in a wide variety of cells [16]. Our results show that at a low MOI, lentivirus achieved a high infection in a variety of human lung cancer cells. Lentivirus is the more feasible delivery system for use *in vivo* or for clinical applications in lung cancer gene therapy.

For clinical usefulness, because of the individual differences between patient tumors, we sought to define a universal and efficient viral vector to deliver a therapeutic gene into multiple types of lung cancer cells. Thus, we used eleven lung cancer cell lines to compare the transduction efficiency of 5 serotypes of AAV with lentivirus. Each cell line represents different classes of lung cancer patients. Our cell line data indicates that lentivirus has the potential for delivering a therapeutic gene into 90% of (10/11) lung cancer patients classified as SCLC (classical and variant), large cell carcinoma, and carcinoid.

## Conclusions

In conclusion, this study provides valuable information on choosing an effective viral delivery system for future gene therapy studies targeting human lung cancer cells. The ability of the lentivirus to readily infect a human lung cancer cell is due to the VSV glycoprotein. Therefore, the use of lentivirus armed with a therapeutic gene would be the optimal choice for delivery into various types of human lung cancer as an alternative treatment option.

## Materials and methods

### Production of different serotypes of rAAV

The AAV vector plasmid, pAAV2-EGFP (Agilent Technologies) harbors a CMV driven- enhanced GFP gene expression cassette flanked by inverted terminal repeats (ITRs). pAAV-RC plasmid harbors the AAV2 *rep* and *cap* genes encoding the replication and virus capsid structural protein. The pHelper plasmid contains the essential subset of adenovirus genes, VA, E2A and E4 necessary for AAV production in the AAV-293 cells (Agilent Technologies). For AAV2/2 production, the three AAV plasmids (10 ug of each plasmid) were co-transfected into AAV-293 cells using the CalPhos Mammalian transfection kit (Clontech, Mountain View, CA). For AAV2/1 and AAV2/5 production, two plasmids were used, pAAV2-EGFP and the pDP1rs or pDP5rs (Aldevron Fargo, North Dakota) which contains AAV2 *rep* and AAV1 *cap* gene or AAV5 *cap* gene and a subset of adenovirus genes, VA, E2A and E4 necessary for AAV production in AAV-293 cells. AAV-293 cells were transfected with the two plasmids at a ratio of 3:1 pAAV2-EGFP and pDP using the CalPhos Mammalian transfection kit to produce the AAV2/1 or AAV2/5 vectors. The medium was removed and replaced with 15 ml of fresh medium 6 hours after transfection and the plates returned to the 37°C incubator for an additional 72 hours. The transfected cells and the culture medium were transferred into 50-mL conical tubes and the cells were spun down at 500×G for 10 min at 4°C. The supernatant was discarded and the cell pellets were resuspended in 1X PBS buffer. The cell suspension was subjected to 4 rounds of freeze/thaw lysis by switching the tubes between a dry ice-ethanol bath and a 37°C water bath. The cell debris was removed by centrifugation at 1000×G for 10 minutes and then the supernatant was collected. The supernatant containing the crude viral particles were pelleted by high speed centrifugation at 100,000×g for 16 hr at 4°C. Following centrifugation, the pellet was resuspended by vigorous agitation in 5 ml of DNase buffer (10 mM Tris.Cl, pH7.5, 10 mM MgCl_2_) followed by addition of 1,000 units of DNase I and incubation for 1 hr at 37°C. To inactivate the DNase I, 250 μL of 0.5 M EDTA was added and the debris was removed by centrifugation at 10,000×g for 2 min. The supernatant was loaded onto a two-tier CsCl gradient (1.25 g/ml and 1.50 g/ml) and spun at 35,000 rpm for 2 h at 16°C in an SW40 rotor (Beckman Instruments, Palo Alto, CA). The band that contained the viral particles was collected and dialyzed in a Slide-A-Lyzer cassette (Pierce, Rockford, IL) to desalt the virus fraction with PBS buffer. The supernatant was harvested and contained the desalted virus particles. The AAV viruses were stored at −80°C. All AAV and lentivirus production was done in accordance with Institutional Biosafety Committee (IBC) approval from the Research Institute for Children at Children's Hospital, New Orleans, LA.

The AAV2/1, AAV2/4, AAV2/5, and AAV2/8 viral vectors carrying the CMV promoter-driven mCherry transgene were obtained from the Gene Transfer Vector Core Lab, University of Iowa (Iowa City, IA).

### Production of lentivirus

Lentiviral particles pseudotyped with the VSV-G glycoprotein were generated by calcium phosphate-mediated transfection of 293FT cells. 293 FT cells were seeded in 150-cm^2^ plates. Twenty four hours later, the medium was removed and the fresh medium with 25 uM chloroquine (Sigma-Aldrich) was added. The lentiviral vector, packaging, and envelope glycoprotein plasmids were transfected into 293FT cells with the CalPhos Mammalian transfection kit (Clontech, Mountain View, CA) according to the manufacturer’s instruction. The amount of DNA used per plate was 20 μg of lentiviral vector plasmids pNL-CMV/EGFP/WPREΔU3 or pNL-CMV/mCherry/WPRE ΔU3, 13 μg of packaging plasmid pCD/NL-BH*ΔΔΔ, and 6.5 μg pLTR-G. All plasmids were a kind gift from Jakob Reiser, FDA, Silver Spring, MD. The medium was removed 12 hr after transfection and replaced with 15 ml of fresh culture medium. Forty eight hours post transfection, the vector-containing medium was harvested. The harvested medium was centrifuged at 500 × *g* for 5 min and filtered through a 0.45-μ pore size filter (Pall Co., Ann Arbor, MI) to remove the cell debris. For vector concentration by ultracentrifugation, the filtered vector-containing medium was transferred into high speed centrifuge tubes (Beckman Coulter, Fullerton, CA) and centrifuged for 2 h at 25,000 rpm at 4°C using a Beckman SW32Ti ultracentrifuge rotor (Beckman Coulter, Fullerton, CA). The resulting pellet was resuspended in 100 μl of PBS for 2 h at 4°C. Virus aliquots were stored at −80°C.

### Viral titer measurement

For the lentivirus, the viral titers were determined by using flow cytometry analysis (BD Biosciences). Hela cells were infected with a serial dilution from 10^0^ to 10^5^ of the lentivirus containing CMV promoter driven fluorescent gene mCherry or EGFP in Dulbecco’s modified Eagle’s medium with 8 ug/mL polybrene (Sigma-Aldrich, Milwaukee, WI). Twenty four hours post-infection, the medium was removed and replaced with fresh medium without polybrene. Forty eight hours later, the supernatant was discarded and the Hela cells were harvested. The cells were fixed with 4% paraformaldehyde buffer and the proportion of infected cells was detected by flow cytometry analysis to determine the titer.

For AAV virus, the viral titer was measured using HT-1080 cells. A serial dilution of the AAV virus was added to the HT1080 cells. After 2 hours, fresh medium was added to the cells and then incubated at 37°C for an additional 48 hours. The proportion of AAV infected cells were determined by flow cytometry analysis.

### Cell lines and culture

The cells used for virus production, 293FT cells (Life Technologies) were cultured in Dulbecco’s modified Eagle’s medium (DMEM, high-glucose) supplemented with 10% FBS, 1% Glutamine, 1 mM sodium pyruvate, 0.1 mM non-essential amino acids, 1X Pen/Strep (10,000 IU penicillin and 10,000 ug/ml streptomycin), with 500 ug/mL geneticin (Mediatech, Inc., Manassas, VA). AAV-293, HT1080 (Agilent Technologies, La Jolla, CA) and Hela cells (ATCC) were cultured in Dulbecco’s modified Eagle’s medium (DMEM, high-glucose) with 10% fetal calf serum (Atlanta Biological Inc., Norcross, Georgia), 1X Pen/Strep (10,000 IU penicillin and 10,000 ug/ml streptomycin) (Mediatech, Inc., Manassas, VA.) in a 5% CO2 incubator at 37°C.

The human SCLC cell lines, DMS 53, NCI-H69, NCI-H82, NCI-H209, NCI-H510 and SHP-77, as well as NSCLC cell lines, NCI-H23 (adenocarcinoma), NCI-H661, NCI-H460 and NCI-H1155 (large cell lung cancer), NCI-H727 (carcinoid) were obtained from the American Type Culture Collection, Manassas, VA and cultured in RPMI 1640 medium (Mediatech, Inc., Manassas, VA.) supplemented with 10% fetal calf serum (Atlanta Biological Inc., Norcross, Georgia), 1X Pen/Strep (Mediatech, Inc., Manassas, VA.) in a 5% CO2 incubator at 37°C.

## Abbreviations

AAV: Adeno-associated virus; VSV-G: Vesicular stomatitis virus glycoprotein; EGFP: Enhanced green fluorescent protein; SCLC: Small cell lung cancer; NSCLC: Non-small cell lung cancer; MOI: Multiplicity of infection; ITR: Inverted terminal repeat; FGFR-1: Fibroblast growth factor receptor-1; kDA: Kilodalton; RC: Replication and capsid; PBS: Phosphate buffered saline; MgCl2: Magnesium chloride; pen/strep: Penicillin/ streptomycin; EDTA: Ethylenediaminetetraacetic acid; Hr: Hours; Rpm: Revolution per minute.

## Competing interests

No authors have any competing interests to declare.

## Authors’ contributions

CC made the lentivirus and AAV2, AAV1, and AAV5 virus preparations, performed the infections of the various lung cancer cell lines and acquired and analyzed the data generated for the manuscript. VA helped with the cell line cultures and generated the mCherry lentivirus transgene construct. JB assisted in the preparation of the AAV viruses. MSL was involved in the conception, design, and analysis of the data in the manuscript as well as being involved in the critical review of the manuscript. MBB was involved in the conception, design, analysis, and interpretation of the data presented in the manuscript as well as being involved with the revision and critical review of the final version of the manuscript. All authors read and approved the final manuscript.
